# Chromogranin A Cell Density as a Diagnostic Marker for Lymphocytic Colitis

**DOI:** 10.1007/s10620-012-2249-6

**Published:** 2012-06-15

**Authors:** Magdy El-Salhy, Doris Gundersen, Jan G. Hatlebakk, Trygve Hausken

**Affiliations:** 1 Section for Gastroenterology, Department of Medicine, Stord Helse-Fonna Hospital, Box 4000, 54 09 Stord, Norway; 2 Department of Research, Helse-Fonna, Haugesund, Norway; 3 Section for Gastroenterology, Institute of Medicine, University of Bergen, Bergen, Norway

**Keywords:** Diagnosis, Chromogranin A, Colon, Computer image analysis, Immunohistochemistry, Lymphocytic colitis

## Abstract

**Background:**

Lymphocytic colitis (LC) can be mistakenly diagnosed as irritable bowel syndrome (IBS). In a previous study on IBS, some patients showed extremely high colonic chromogranin A cell density. Further examination of these patients showed that they suffered from LC.

**Aims:**

To investigate whether chromogranin A cell density is increased in LC patients and to examine the possibility of using this increase as a marker for the diagnosis of LC.

**Methods:**

Fifty-seven patients diagnosed with LC and 54 controls were included in the study. Biopsies from the right and left colon were obtained from both patients and controls, which were immunostained using the Avidin–biotin-complex method for chromogranin A, and cell density was quantified.

**Results:**

In both the right and left colon of patients with LC, the density of chromogranin A was significantly higher than in controls. This increase in chromogranin A cells occurs whether the number of these cells is expressed as number/mm^2^ epithelium or as number/field. Chromogranin A cell density for the right and left colon expressed as number of cells/mm^2^ epithelium or as cell number/field showed a high sensitivity and specificity as a diagnostic marker for LC.

**Conclusions:**

Chromogranin A is a common marker for endocrine cells, and the present finding suggests that colonic hormones are involved in the pathophysiology of LC. The chromogranin cell density seems to be a good diagnostic marker with high sensitivity and specificity in both the right and left colon, thus sigmoidoscopy can be used in the diagnosis of LC using with this marker.

## Introduction

Microscopic colitis (MC) is a chronic condition, which is characterized by watery diarrhea with normal radiologic and endoscopic findings. However, histopathological examinations of the colon reveal abnormal histology [1], which is of two distinctive types: lymphocytic colitis (LC) and collagenous colitis (CC) [1]. LC exhibits an increased number of colonic intra-epithelial lymphocytes (>20/100 epithelial cells), increased inflammatory cells within lamina propria, and preserved crypt architecture [1]. Population-based studies in Europe and in the United States have reported an incidence of LC ranging from 3.1 to 9.8 per 100,000 [2–10]. The prevalence of LC in Europe and the United States in population-based investigation has been found to be 14.2 per 100,000 population [2–9].

LC and irritable bowel syndrome (IBS) have similar symptoms and a normal endoscopic appearance, as well as normal radiologic findings [10, 11]. Several studies have shown that LC can be mistakenly diagnosed as IBS [12, 13]. In a study on the colonic chromogranin A cell density in IBS patients [13], nine patients out of 50 showed extremely high colonic chromogranin A cell density [13]. This high density was in contrast to the low density of chromogranin cells in the rest of the IBS patients studied [13]. Further examination of these nine patients showed that they suffered from LC, although they fulfilled the Rome III criteria for IBS [13].

The symptoms of LC suggest an abnormally rapid intestinal motility and a decrease in the absorption of water. The neuroendocrine system of the gut plays a significant role in regulating gut motility, as well as the absorption of water and salts [14–17]. It is reasonable to assume, therefore, that colonic endocrine cells may be affected, and may be involved in the pathophysiology of this disorder. Chromogranin A is a 68-kDa protein comprising 439 amino acid residues, which was isolated for the first time from secretory granules of the bovine adrenal medulla [18]. Chromogranin A is co-stored and co-released with monoamines and peptide hormones of the adrenal medulla, pituitary gland, parathyroid, thyroid C-cells, pancreatic islets, endocrine cells of the gastrointestinal tract, and sympathetic nerves [19–20]. Therefore, chromogranin A is considered to be a general marker for all endocrine cells.

The current study was undertaken to examine the colonic chromogranin cell density in a patient cohort with LC in order to establish whether a higher density of these cells occur, and to further examine the possibility of using colonic chromogranin A as a marker for the diagnosis of LC.

## Materials and Methods

### Patients and Controls

Fifty-seven patients diagnosed with LC during the period from 2007 to 2010 in all three hospitals of the Helse-Fonna region in western Norway, namely, Stord, Haugesund, and Odda hospitals, were included in this study. These patients included 41 females and 16 males, with an average age of 49 years (range 19–84 years). Fifty-four subjects that underwent colonoscopy with biopsies were used as controls. These subjects underwent colonoscopy because of gastrointestinal bleeding, where the source of bleeding was identified as hemorrhoids (26) and angiodysplasia (8), and a further 20 subjects were tested because of health worries caused by a relative being diagnosed with colon carcinoma. The control group was comprised of 38 females and 16 males, with an average age of 49 years (range 18–67 years).

The study was performed in accordance with the Declaration of Helsinki and was approved by the local Committee for Medical Research Ethics. All subjects gave oral and written consent.

### Colonoscopy

Colonoscopy was performed in patients and controls and two biopsies were taken from the cecum, from both the ascending colon and the right half of the transverse colon. All biopsies were pooled together and used as right colon, regardless of gender. In addition, two biopsies were taken from the left half of transverse colon, from the descending colon, and from the sigmoid colon. These six biopsies were pooled together and used as left colon.

### Histopathology and Immunohistochemistry

Biopsies were fixed in 4 % buffered paraformaldehyde overnight, embedded in paraffin and cut into 5-μm-thick sections. The sections were stained by hematoxylin and eosin (H&E) and immunostained with the avidin–biotin-complex (ABC) method using the Vectastain ABC-kit (Vector Laboratories) as described previously [21]. The primary antibody used was a monoclonal mouse anti-N-terminal of purified chromogranin A (DakoCytomation, code no. M869). The antibody was used at dilutions of 1:1,500. The second layer, biotinylated mouse anti-IgG was obtained from DakoCytomation. Negative and positive controls were the same as those described previously [21].

### Computerized Image Analysis

The number of chromogranin A immunoreactive cells and the area of the epithelial cells was measured using Olympus software: Cell ^D. When using 40× objectives, the frame (field) on the monitor represented an area of 0.14 mm^2^ of the tissue. Measurements were performed in ten randomly chosen fields for each individual. The immunostained sections of patients and controls were coded and mixed, and measurements were made without the knowledge of sections identity. The 40× objective was used. The data from fields were tabulated, the number of cells/mm^2^ of epithelium was computed, and statistical analysis was performed automatically. The number of chromogranin A immunoreactive cells was also counted per microscopic field in 10 fields for each individual.

### Statistical Analysis

As the results of controls and patients passed the normality test, the *t* test was performed. *p* < 0.05 was considered to be statistically significant.

## Results

### Endoscopy, Histopathology, and Immunohistochemistry

The colons of patients and control subjects were shown to be macroscopically normal. Histopathological examination of the colon biopsies from controls revealed normal histology and those from patients showed typical LC histopathology.

### Computerized Image Analysis

The chromogranin A cell density in the right colon of the controls was 21.5 ± 0.5 per mm^2^ epithelium (mean ± SE), and in the left colon was 27.2 ± 1.4 per mm^2^ epithelium. Chromogranin A cell density was significantly higher in the right colon than in the left (*p* = 0.02). The number of chromogranin A cells per field in the right colon of controls was 9.3 ± 0.5 and in the left colon was 8.9 ± 0.6. There was no significant difference between right and left colon regarding the number of chromogranin A cells per field (*p* = 0.3). The chromogranin A cell density in the right colon of patients with LC was 68.8 ± 4 per mm^2^ epithelium, and in the left colon was 87 ± 4 per mm^2^ epithelium. The cell density of chromogranin A was higher in the left colon than in the right colon of patients with LC (*p* = 0.002). In patients with LC, the number of chromogranin A cells per field in the right colon was 38.7 ± 2 and in the left colon was 36.6 ± 2. There was no significant difference in the number of chromogranin A cells per field between right and left colon in patients with LC.

**Fig. 1:**
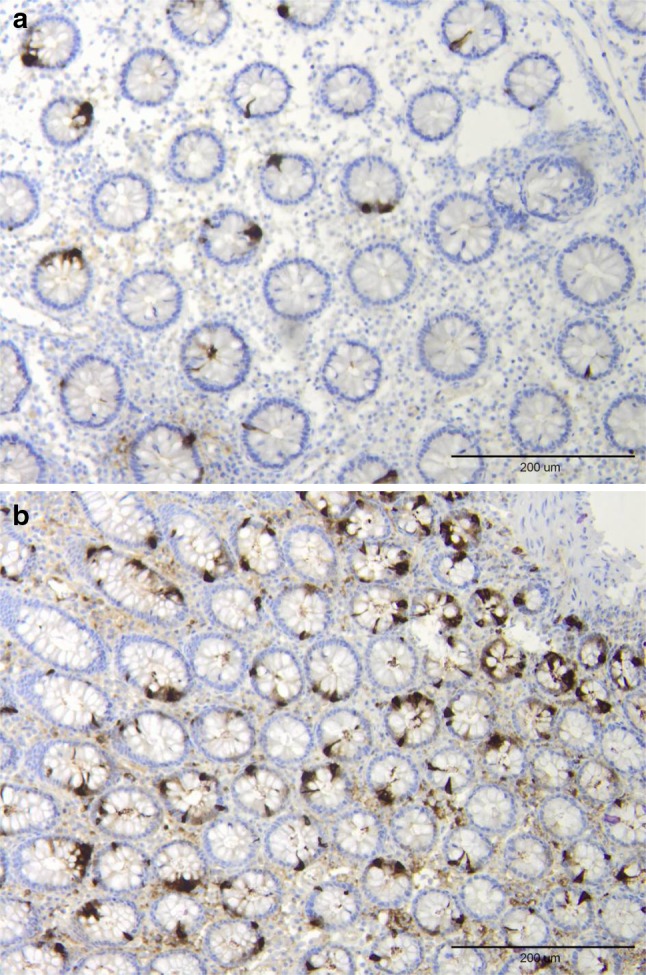
Chromogranin A-immunoreactive cells in the colon of a control (**a**) and a patient with lymphocytic colitis (**b**)

**Fig. 2:**
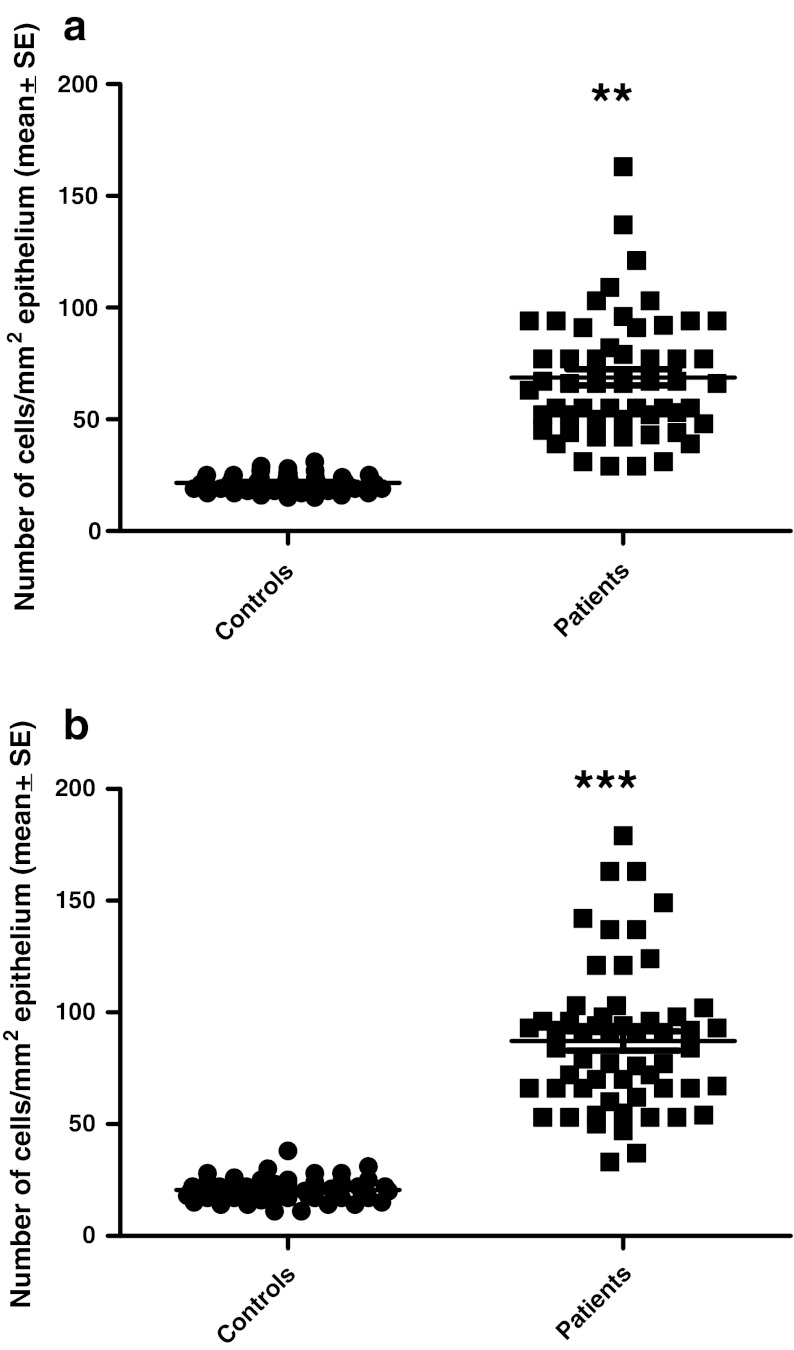
Chromogranin A cell density in the controls and patients with lymphocytic colitis as expressed per mm^2^ epithelium: in the right colon (**a**) and left colon (**b**). **p < 0.01, ***p < 0.001

**Fig. 3:**
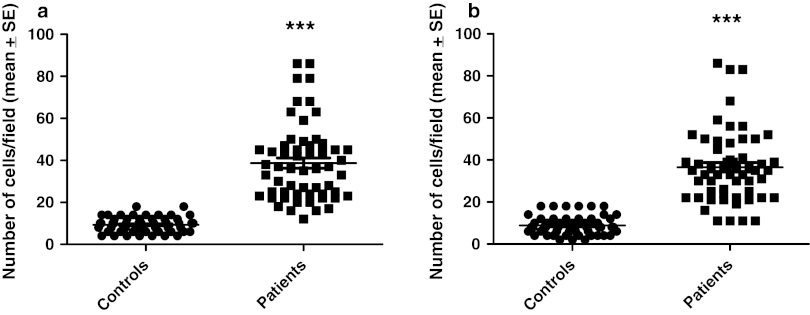
Chromogranin A cell density expressed as he number of cells per field in controls and patients with lymphocytic colitis: in the right colon (**a**) and left colon (**b**). *Symbols* are the same as in Fig. 2

In both the right and left colon of patients with LC, the density of chromogranin A was significantly higher than in controls (Fig. 1). The increase in chromogranin A cells in patients with LC occurred whether the number of these cells was expressed as number/mm^2^ epithelium or as number/field (Figs. 2, 3).

**Fig. 4:**
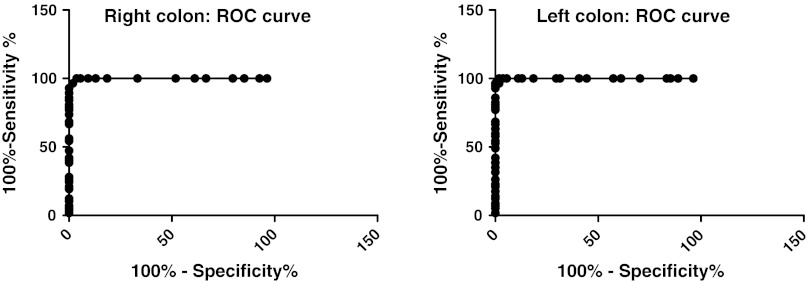
Receiver-operator characteristic (ROC) curve for chromogranin A cell density for the right (**a**) and left colon expressed as number of cells/mm^2^ epithelium (**b**)

Receiver-operator characteristic (ROC) curves for chromogranin A cell density for the right and left colon show that in the right colon at a cut-off value of >30 cells/mm^2^ epithelium, the sensitivity is 97 % and specificity is 98 %. In the left colon, at a cut-off of >29 cells/mm^2^ epithelium the sensitivity is 100 % and specificity is 94 % (Fig. 4).

**Fig. 5:**
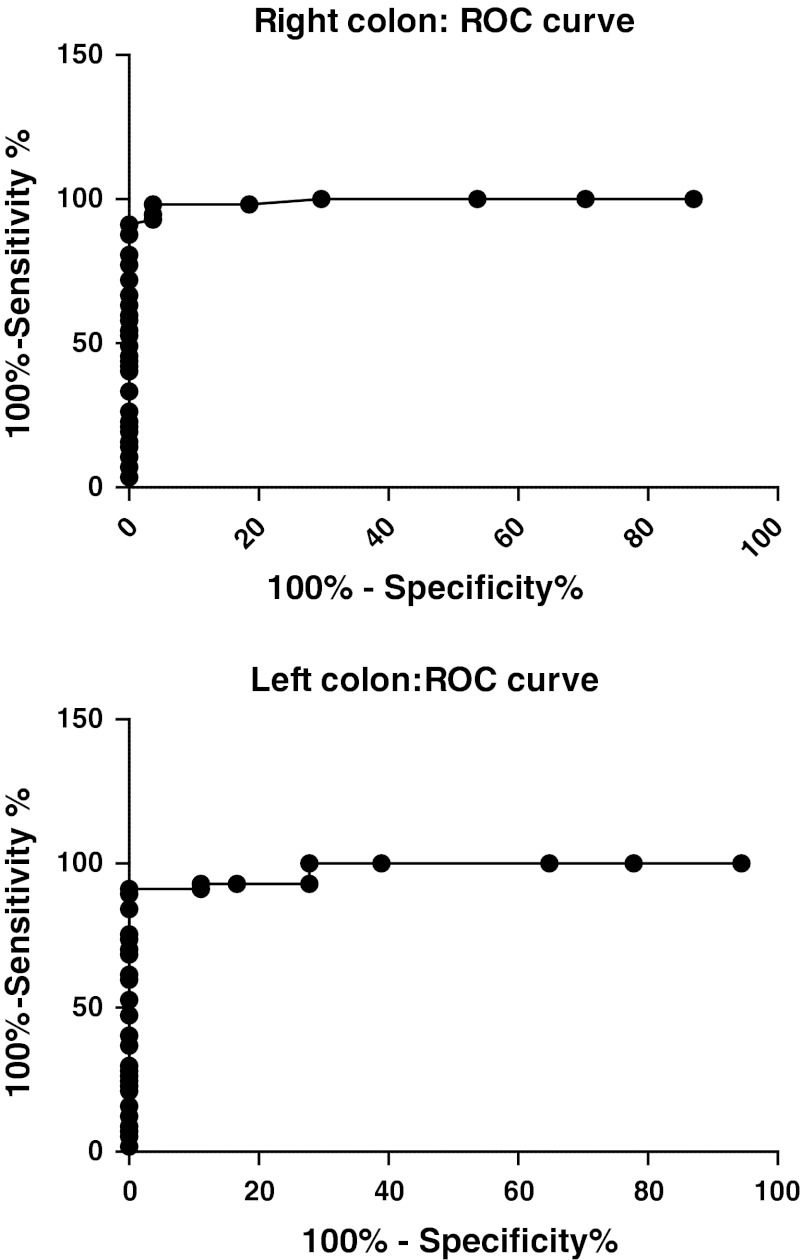
ROC curve chromogranin A cell density in the right colon (**a**) and left colon (**b**), as expressed number of cells per field

ROC curves for chromogranin A cell number/field for the right colon shows that at a cut-off of >15 cells/field, the sensitivity is 98 % and specificity is 96 %. In the left colon, at cut-off values of >15 cells/field, the sensitivity is 93 % and specificity is 89 % (Fig. 5).

## Discussion

The chromogranin A cell density was significantly higher in the right colon than in the left colon when the number of cells was counted with regards to epithelium cell numbers, but not when it was analyzed by microscopic field. In patients with LC, it was the opposite, where the left colon had a higher density of chromogranin cells. Again, there was no difference in chromogranin cell density between the right and left colon when the cells were analyzed per microscopic field. However, the significant difference obtained here could be a statistical type I error. The difference between the outcome when the number of chromogranin cells was compared to epithelium or microscopic field could be due the fact that chromogranin cells, which are situated among the epithelial cells, are more counted with regards to epithelium.

As chromogranin A is a common marker for endocrine cells, the current finding of a high density of colonic chromogranin A cells in patients with LC suggests that colonic hormones are involved in the pathophysiology of LC. Several studies present solid evidence for the interaction of the gut neuroendocrine peptides/amines and the local immune system in the gut. This is referred to as the endocrine/immune axis [22]. Thus, chromogranin-derived peptides, such as chromofugin and vasostatin-I, are able to penetrate into polymorphonuclear neutrophils, inducing an extracellular calcium entry [23]. This study illustrates the role of chromogranins in active communication between the neuroendocrine and immune system. Moreover, the chromogranin-derived peptide, catestatin stimulates chemotaxis of human peripheral blood monocytes [24, 25]. Secreoneurin, a chromogranin-derived peptide, reduces IL-6 release from eosinophils [26]. In addition, chromogranin-derived peptides modulate the endothelial permeability during inflammatory processes. Chromogranin A prevents the vascular leakage induced by the tumor necrosis factor (TNF)-alpha in a mouse model [26]. It is noteworthy that colonic chromogranin A cells have been found to increase in patients with ulcerative colitis (UC) and Crohn’s disease (CD) [27]. Furthermore, elevated plasma and serum chromogranin levels have been reported in UC and CD patients [28, 29].

The chromogranin A cell density seems to be a good diagnostic marker with a high sensitivity and specificity. This was shown to be true whether biopsies were taken from the right or left colon. Furthermore, chromogranin A cell density showed the same high specificity and sensitivity when it was measured as number of cells per microscopic field. This facilitates the use of this marker even in small laboratories that are not equipped with computer image analysis facilities. The diagnosis of LC is based mainly on the finding of intraepithelial lymphocytosis. Lymphocytic infiltration has been reported, however, to occur more often in the right than in left colon [30]. In another study, biopsies taken from the left colon (sigmoid or descending colon) were enough to make the diagnosis in 98.6 % of cases [30]. Thus, the reliability of using flexible sigmoidoscopy in making the diagnosis LC is not clear, and remains controversial. Chromogranin A cell density as a marker showed almost the same high sensitivity and specificity in biopsies taken from right or left colon, making sigmoidoscopy suitable for use with this marker.
